# Targeting 4-1BB and PD-L1 induces potent and durable antitumor immunity in B-cell lymphoma

**DOI:** 10.3389/fimmu.2022.1004475

**Published:** 2022-12-05

**Authors:** Yichen Wang, Xuyao Zhang, Caili Xu, Yanyang Nan, Jiajun Fan, Xian Zeng, Byoung S. Kwon, Dianwen Ju

**Affiliations:** ^1^ School of Pharmacy and Minhang Hospital, Shanghai Engineering Research Center of Immunotherapeutics, Fudan University, Shanghai, China; ^2^ Eutilex Institute for Biomedical Research, Eutilex Co., Ltd, Seoul, South Korea; ^3^ Department of Biologics, Fudan Zhangjiang Institute, Shanghai, China

**Keywords:** B-cell lymphoma, anti-PD-L1 ICI, 4-1BB agonist, CD8+ T cells, combination therapy

## Abstract

**Introduction:**

Although PD-1/L1 mAb has demonstrated clinical benefits in certain cancer types, low response rate and resistance remain the main challenges for the application of these immune checkpoint inhibitors (ICIs). 4-1BB is a co-stimulator molecule expressed in T cells, which could enhance T cell proliferation and activation. Herein, the synergetic antitumor effect and underlying mechanism of 4-1BB agonist combined with PD-1/PD-L1 blockade were determined in B-cell lymphoma (BCL).

**Methods:**

Subcutaneous transplantation BCL tumor models and metastasis models were established to evaluate the therapeutic effect of PD-L1 antibody and/or 4-1BB agonist in vivo. For the mechanistic study, RNA-seq was applied to analyze the tumor microenvironment and immune-related signal pathway after combination treatment. The level of IFN-γ, perforin, and granzyme B were determined by ELISA and Real-time PCR assays, while tumor-infiltrating T cells were measured by flow cytometry and immunohistochemical analysis. CD4/CD8 specific antibodies were employed to deplete the related T cells to investigate the role CD4+ and CD8+ T cells played in combination treatment.

**Results:**

Our results showed that combining anti-PD-L1 ICI and 4-1BB agonists elicited regression of BCL and significantly extended the survival of mice compared to either monotherapy. Co-targeting PD-L1 and 4-1BB preferentially promoted intratumoral cytotoxic lymphocyte infiltration and remodeled their function. RNA-sequence analysis uncovered a series of up-regulated genes related to the activation and proliferation of cytotoxic T lymphocytes, further characterized by increased cytokines including IFN-γ, granzyme B, and perforin. Furthermore, depleting CD8+ T cells not CD4+ T cells totally abrogated the antitumor efficacy, indicating the crucial function of the CD8+ T cell subset in the combination therapy.

**Discussion:**

In summary, our findings demonstrated that 4-1BB agonistic antibody intensified the antitumor immunity of anti-PD-1/PD-L1 ICI via promoting CD8+ T cell infiltration and activation, providing a novel therapeutic strategy to BCL.

## Introduction

Although PD-1/PD-L1 axis-targeting immune checkpoint inhibitor (ICI) has revolutionized the therapeutic modality of several types of cancers, an obvious fraction of cancer patients showed no responses to ICI monotherapy (1, 2). Multiple factors such as immune cell infiltration, expression of immune checkpoint and cytokine signaling could monitor the response to ICIs and influence their efficacy (3, 4). Elucidation of the underlying immunologic characteristic of the tumor microenvironment (TME) associated with resistance will benefit the patients treated with ICI monotherapy and reveal the determinants for ICIs combination therapy.

As one prominent co-stimulator, 4-1BB is mainly expressed on natural killer T and CD4^+^/CD8^+^ T cells (5–7). Upon conjunction with soluble 4-1BBL or agonistic monoclonal antibody (mAb), 4-1BB forms a heterotrimer and induces T-cell proliferation, cytokine release, and upregulation of antiapoptotic molecules (8). In 4-1BB-deficient models, the function of cytotoxic T lymphocytes is mostly diminished, and 4-1BB on infiltrated T cells in TME could serve as a marker to predict the antitumor effect of immunotherapy (9). A comprehensive study revealed for the first time that targeting 4-1BB has strong antitumor effects *via* injecting mice bearing Ag104A sarcoma and P815 mastocytoma with anti-4-1BB mAbs (10). Kinetic studies of 4-1BB expression on T cells indicate that although mechanisms for the differential costimulatory ability are not completely elucidated in T cell subsets, 4-1BB is an ideal target on CD8^+^ T cells for immunotherapy. Indeed, while agonic mAb of 4-1BB could reduce the tumor size and increase survival in multiple preclinical studies (11–13), only limited clinical benefit has been observed due to dose-dependent hepatic toxicity (14–16).

Anti-4-1BB mAb has been proved as an enhancer in the antitumor immunity (17). The bispecific Ab targeting 4-1BB and HER2 showed potent therapeutic effects in HER2 positive cancer (18). IL-12 and 4-1BB were found to possess synergistic antitumor effect *via* activating CD8^+^ T cell response in mouse models (19). As to the impact of 4-1BB on the therapeutic effect of ICIs, researches demonstrated that costimulatory pathways and immune checkpoint pathways were interdependent (20, 21). Lack of the costimulatory signals led to increased PD-1 expression, which further decreased IL-2 receptors that were necessary for T cell proliferation, whereas 4-1BB co-stimulation potently enhanced reinvigoration of infiltrated T cells in TME (22). Although these findings imply a potential synergy between PD-1 blockade and 4-1BB agonist, the synergistic antitumor effect between PD-L1 blockade and 4-1BB agonist remains unclear (23).

In this context, antitumor immunity of PD-1/PD-L1 blockade and 4-1BB agonist was evaluated in immunocompetent B-cell lymphoma (BCL) models. The transcriptional profile was further analyzed to uncover the underlying mechanisms. Our results highlighted PD-L1 blockade combined with 4-1BB agonist as a potential therapeutic strategy for BCL.

## Results

### Anti-PD-L1 mAb and 4-1BB agonist elicited synergistic antitumor activity

**Figure 1:**
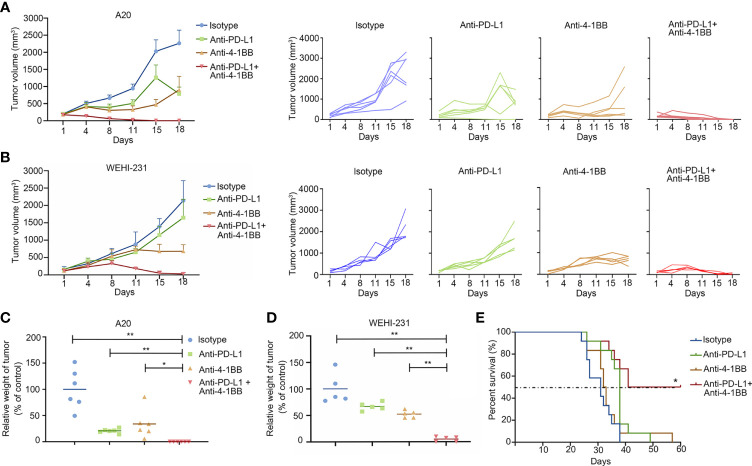
4-1BB agonism and PD-L1 blockade elicited synergistic antitumor effect in B-cell lymphoma. **(A, B)** The subcutaneous A20 tumor model (n = 6) or WEHI-231 tumor model (n = 5) were well-established in BALB/c mice. Tumor volume was measured twice a week. Treatment was initialized when the tumor volume reached 100 mm^3^. **(C, D)** Tumor weight was measured at the end of the treatment. **(E)** The metastatic A20 model was established to evaluate the antitumor effect on survival (n = 10). * means p-value < 0.05, and ** means p-value < 0.01.

To investigate the feasibility of dual-targeting PD-L1 and 4-1BB in BCL tumors, mice implanted with A20 or WEHI-231 tumors were treated with 4-1BB agonist, anti-PD-L1 mAb, 4-1BB agonist combined with anti-PD-L1 mAb, respectively. As shown in **Figures 1A, B**, compared with an equivalent dose of ICI alone, the combined therapy showed a more potent anti-tumor effect in both well-established A20 and WEHI-231 models. A20 tumor-bearing mice were sacrificed on day 18. All mice in the combined group were tumor-free, and the relative tumor weight compared to control in the groups of anti-PD-L1 and 4-1BB agonist antibody were 20.7 ± 4.1% and 33.9 ± 27.5% respectively (**Figure 1C**). Similar results were observed in WEHI-231 model. Compared with the group under monotherapy with anti-PD-L1 or 4-1BB agonist, tumor volume in the combined group began to decrease on day 8 and continued until the end of treatment (**Figure 1B**). Relative tumor weight in the groups of anti-PD-L1, 4-1BB agonist, anti-PD-L1 plus 4-1BB agonist were 66.8 ± 7.5%, 52.2 ± 7.0% and 5.0 ± 4.6% respectively (**Figure 1D**). To further explore whether the combined therapy could extend the survival, A20 metastatic model was established. Compared with the 4-1BB agonist or anti-PD-L1 monotherapy, combined therapy significantly extended the median survival. Importantly, half of the mice were still vigorous by the end of this experiment (**Figure 1E**). These data showed that anti-PD-L1 therapy combined with 4-1BB agonist elicited potent and durable antitumor effect in subcutaneous and metastatic BCL models.

### Transcriptional profile involved in the synergistic antitumor effect

**Figure 2:**
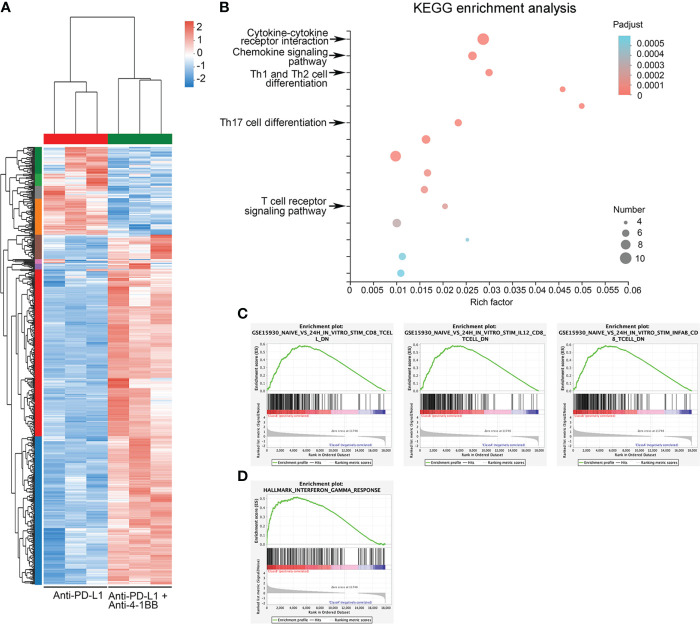
Mechanisms underlying the synergistic antitumor efficacy of the combination therapy. **(A)** Heatmap showed the differentially expressed genes between the anti-PD-L1 group and the combined therapy group. **(B)** Enrichment of KEGG pathway of differentially expressed genes. **(C)** GSEA showed an enrichment of the gene set related to CD8^+^ T cell activation. **(D)** GSEA showed enrichment of activation of IFN-γ response gene sets.

To reveal mechanisms underlying the synergistic antitumor effect, we performed RNA-sequencing on mouse A20 tumor tissue at 10 days post-treatment. In brief, Six tumor-bearing mice were divided equally into two groups and treated with PD-L1 antibody or a combination of 4-1bb agonist and PD-L1 antibody, and their tumor tissues were examined ten days later to analyze the transcriptional profile in the TME. Compared to anti-PD-L1 monotherapy, anti-PD-L1 therapy combined with 4-1BB agonist significantly altered the expression profiles of 538 genes (defined as *P*<0.05, fold change>2) in TME (**Figure 2A**). Furthermore, the biological functions of the modules were analyzed by KEGG (**Figure 2B**). Interestingly, most of the increased genes were associated with classical antitumor immunity signal pathways, including cytokine and chemokine pathways (1^st^ and 2^nd^), and differentiation of Th1, Th2, and Th17 cells (3^rd^) (adjusted *P*<0.0005). In addition, there were enrichments in Protein digestion and absorption (4^th^), ECM-receptor interaction (5^th^), NF-kappa B signaling pathway (7^th^), PI3K-Akt signaling pathway (8^th^), and Cell adhesion molecules (9^th^), demonstrating that the combination therapy had a widespread influence on the metabolism and metastasis of tumor cells (**Figure 2B**). Integrally, GSEA analysis showed that combined therapy increased T cell activation and IFN-γ signal pathway (**Figures 2C, D**). These results uncovered the transcriptional landscape in BCL tumors under the cotreatment with anti-PD-L1 ICI and 4-1BB agonist.

### 4-1BB agonist potentiated anti-PD-L1 mAb-induced T cell immunity

**Figure 3:**
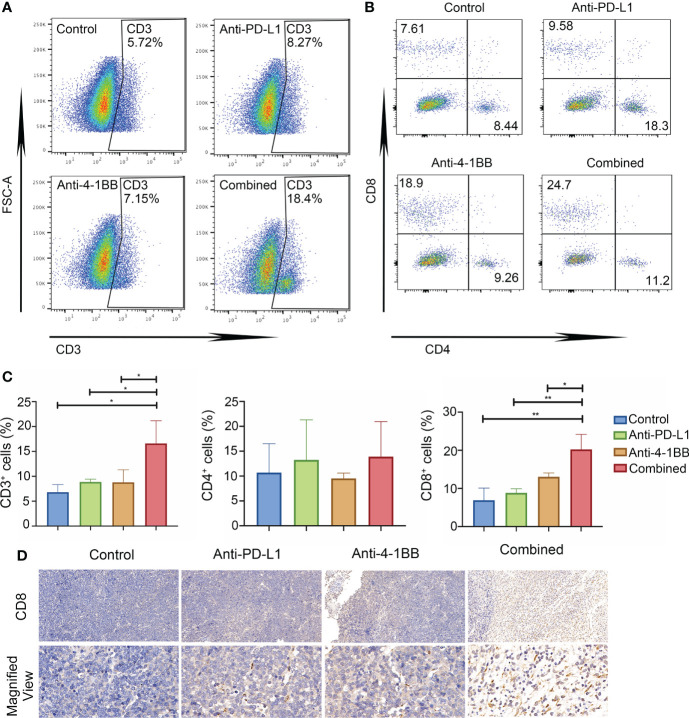
The combined therapy increased cytotoxic T-cell infiltration and expression of PD-L1. **(A–C)** The proportion of CD4/CD8 positive cells in the tumor tissue in each group (n = 3) after one week of treatment. **(D)** The infiltration of CD8^+^ T cell in tumor tissue detected by Immunohistochemistry (IHC) (Magnification × 20 (up), Magnification × 90 (below)). * means p-value < 0.05, and ** means p-value < 0.01.

Next, we sought to detect T cells infiltration in TME *via* flow cytometry. Compared to anti-PD-L1 monotherapy, a significant increase of tumor-infiltrating lymphocyte (TIL) was observed in the group of combinatorial therapy (**Figure 3A**). Furthermore, we determined T cell subsets in the indicated cohorts and found that anti-PD-L1 monotherapy presented modest impact on the infiltration of T cells, whereas there were obvious changes in the combinatorial group with a two-fold increase of CD8^+^ T cells (**Figures 3B, C**). Immunohistochemistry staining was also performed to confirm CD8^+^ T cell infiltration. As shown in **Figure 3D**, compared to anti-PD-L1 alone, PD-L1 blockade plus 4-1BB agonism indeed promoted the infiltration of CD8^+^ T cells. Summary, these data indicated that 4-1BB agonist and PD-L1 blockade resulted in synergistic antitumor efficacy *via* reinforcing T-cell immunity and PD-L1 expression.

### 4-1BB agonist potentiated cytolytic capacity of infiltrated T cells

**Figure 4:**
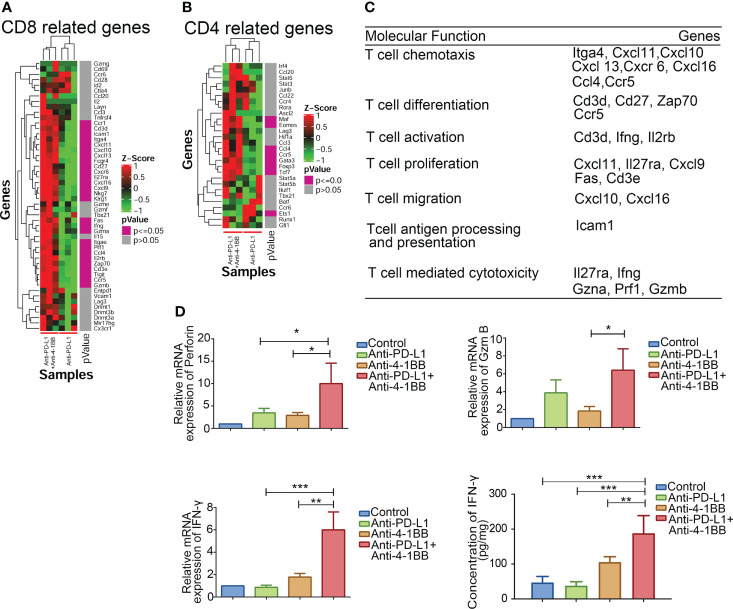
Functions of CD8^+^ T cells in the combination therapy. **(A, B)** Combinational therapy significantly upregulated genes related to CD8^+^ and CD4^+^ T cells. **(C)** Genes were classified and grouped based on their molecular function. **(D)** Perforin, IFN-γ, and granzyme B in tumors were measured by RT-PCR or ELISA after combinational treatment (n = 3, mean ± SD). * means p-value < 0.05, ** means p-value < 0.01, and *** means p-value < 0.001.

Infiltrated T cells in an established TME are mostly dysfunctional. Thus, gene signature and function of the infiltrated T cells were further evaluated after anti-PD-L1 and 4-1BB agonist combined therapy. We examined the genes related to T cell function in TME, and found that the upregulated genes were significantly enriched in T cell chemotaxis, differentiation, proliferation and activation (**Figures 4A–C**). Importantly, compared to anti-PD-L1 monotherapy, transcriptional levels of *ifng*, *gzma*, *prf1* and *gzmb* were also intensified by the combined therapy. To confirm immune-related gene expression signatures associated with response to T cell cytotoxicity, mRNA expression of key factors related to T cell cytotoxicity were also measured *via* RT-PCR and ELISA assay. As shown in **Figure 4D**, 4-1BB signaling significantly potent the release of IFN-γ, perforin and granzyme B from T cells induced by anti-PD-L1 mAb. In summary, these results indicated that 4-1BB agonist in combination with anti-PD-L1 ICI potentiated cytolytic capacity of infiltrating T cells.

### CD8^+^ T cell was essential to the antitumor efficacy elicited by the combined therapy

**Figure 5:**
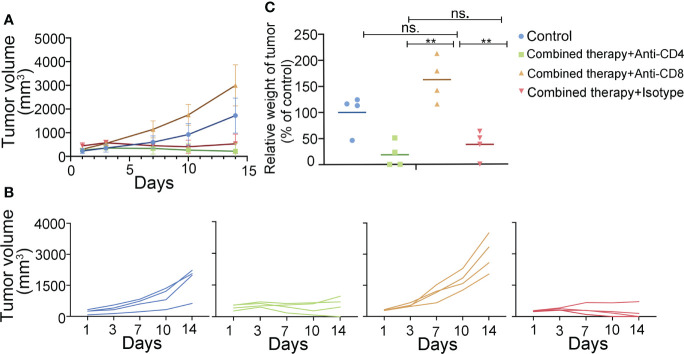
T cell subsets in the antitumor efficacy of anti-PD-L1 mAb combined with 4-1BB agonist. The antitumor effect of the combined therapy was abolished by CD8^+^ T cell depletion. **(A, B)** Tumor volume was measured twice a week. **(C)** Tumor weight was measured at the end of the treatment. (n = 4, mean ± SD). ** means p-value < 0.01. ns means no significance.

To illuminate which T cell subsets were indispensable to the combined therapy-mediated tumor regression, we depleted CD4^+^/CD8^+^ T cells in mice using CD4/8 antibodies, respectively. We then constructed the A20 subcutaneous tumor model and treated the mice with a combination of anti-PD-L1 antibody and 4-1BB agonist. Depleting CD8^+^ T cell completely abolished the therapeutic effect, while CD4^+^ T cell depletion had no obvious effect on the combined therapy (**Figure 5**). The above results confirmed that CD8^+^ T cells played an essential role in the combined therapy.

## Discussion

Considering the fact that PD-1/PD-L1 axis confers cancer cells evasion from hosts’ immune system, digging excellent anti-PD-1/PD-L1 ICIs will inspire patients against malignancies (24). However, only a small subset of patients received benefits from these ICIs, of which the absence and exhaustion of tumor-infiltrated cytotoxic T lymphocytes are the main symptoms (25, 26). As one of the prominent co-stimulators (19, 27), 4-1BB signaling could stimulate T cell proliferation and activation, and enhance the cytotoxicity of adaptive T therapy (28–30). Herein, we determined the synergistic effect of anti-PD-L1 therapy in combination with 4-1BB agonists in BCL.

TIL in tumor environment is considered to be the crucial factor determining the antitumor immunity of PD-1/PD-L1 blockade (31, 32). Various efforts have been made to enhance antitumor immunity of tumor infiltrating lymphocytes. Fusion protein consisting of chemokine CCL4 and collagen-binding domain was applied to recruit TILs to improve the antitumor immunity of anti-PD-L1 monotherapy in multiple tumor models (33). Positive correlation has been uncovered in elevated collagen and exhausted T cells in lung cancers. Reducing tumor collagen deposition could increase infiltration of CD8^+^ T cells and overcome resistance to anti-PD-L1 therapy (34). Physical therapies including thermotherapy and radiation therapy were also reported to promote T cell infiltration and synergize with anti-PD-L1 treatment (35). In addition, activating 4-1BB mAb was found to increase the ratio of tissue-resident T cells in pulmonary and hepatocellular carcinoma (36, 37). Bispecific antibodies MCLA-145 and ABL503 were generated to evaluate the antitumor effect and liver toxicity of co-targeting 4-1BB and PD-L1 (21). Results indicated that high dose of bispecific antibody (10 mg/kg) could induce strong antitumor efficacy with low liver toxicity in MC38 tumor model (38). Our research showed that anti-PD-L1 mAb in combination with low dose of 4-1BB agonistic Ab (1 mg/kg) elicited synergistic antitumor activity with no obvious toxicity, whereas PD-L1 blockade or 4-1BB agonism alone only had modest antitumor effects. Considering the significant discrepancy in dosage, combination therapy targeting 4-1BB and PD-L1 is still a potential option in the clinic.

Analyzing tumor-infiltrating T cells would not only contribute to investigating the T cell subsets, but also provide insights into the function of tumor-specific cytotoxic T lymphocytes. Generally, T cells are essential to eliminate tumor cells and higher cytotoxic T lymphocytes infiltration in tumor correlates with better prognosis (39–42). While characteristics underlying the infiltrated T lymphocytes in TME are still unclear, and functions of TILs in the context of anti-PD-L1 ICI and 4-1BB agonist have not been fully elucidated. Here, we observed that targeting 4-1BB and PD-L1 not only activated T cell immunity, but also promoted recruitment of effector T lymphocytes into TME. We also analyzed the transcriptional profile of TME, and data indicated that anti-PD-L1 mAb in combination with 4-1BB agonist resulted in reinforcing CD8^+^ T-cell immunity *via* cytokine and chemokine signaling pathway. Furthermore, T cell depletion confirmed that the effect of combinational therapy was dependent on CD8^+^ T cells in BCL.

Currently, ICI has elicited promising antitumor effects in several clinical trials. PD-1 monoclonal antibody nivolumab achieved an objective remission rate of 87% in a phase I clinical trial against refractory recurrent classic Hodgkin’s lymphoma (43). In a phase II clinical trial, the objective remission rate reached 68% with overall survival of 6 months (44). However, the efficacy of PD-1 monoclonal antibodies in the treatment of non-Hodgkin’s lymphoma is not satisfactory, with objective remission rates of only 40% and 36% for nivolumab alone in clinical trials for refractory relapsed follicular lymphoma and refractory relapsed diffuse large B-cell tumor (45, 46). Studies have shown that PD-L1 ligands are associated with 9p23-24 gene amplification (47) and that the 9p24 gene has a higher probability of mutation in Hodgkin’s lymphoma cells (48), whereas the gene is rarely altered in non-Hodgkin’s lymphoma cells (47), thus PD-1/PD-L1 blockade has shown better efficacy in the treatment of Hodgkin’s lymphoma.

To further enhance the efficacy of PD-1/PD-L1 monoclonal antibodies in the treatment of B-cell lymphoma, various combination regimens have been proposed, including other immune checkpoint inhibitors, co-stimulatory molecular agonists, and other types of therapeutic antibodies. An objective remission rate of 80% was achieved in clinical trials combining the PD-1 monoclonal antibody pembrolizumab and the CD20 monoclonal antibody rituximab for the treatment of relapsed follicular lymphoma (45). Complete remission rates of 50%-65% were achieved using PD-1 monoclonal antibody nivolumab in combination with brentuximab vedotin, an ADC drug targeting CD30, for refractory relapsed Hodgkin’s lymphoma (49).

**Figure 6:**
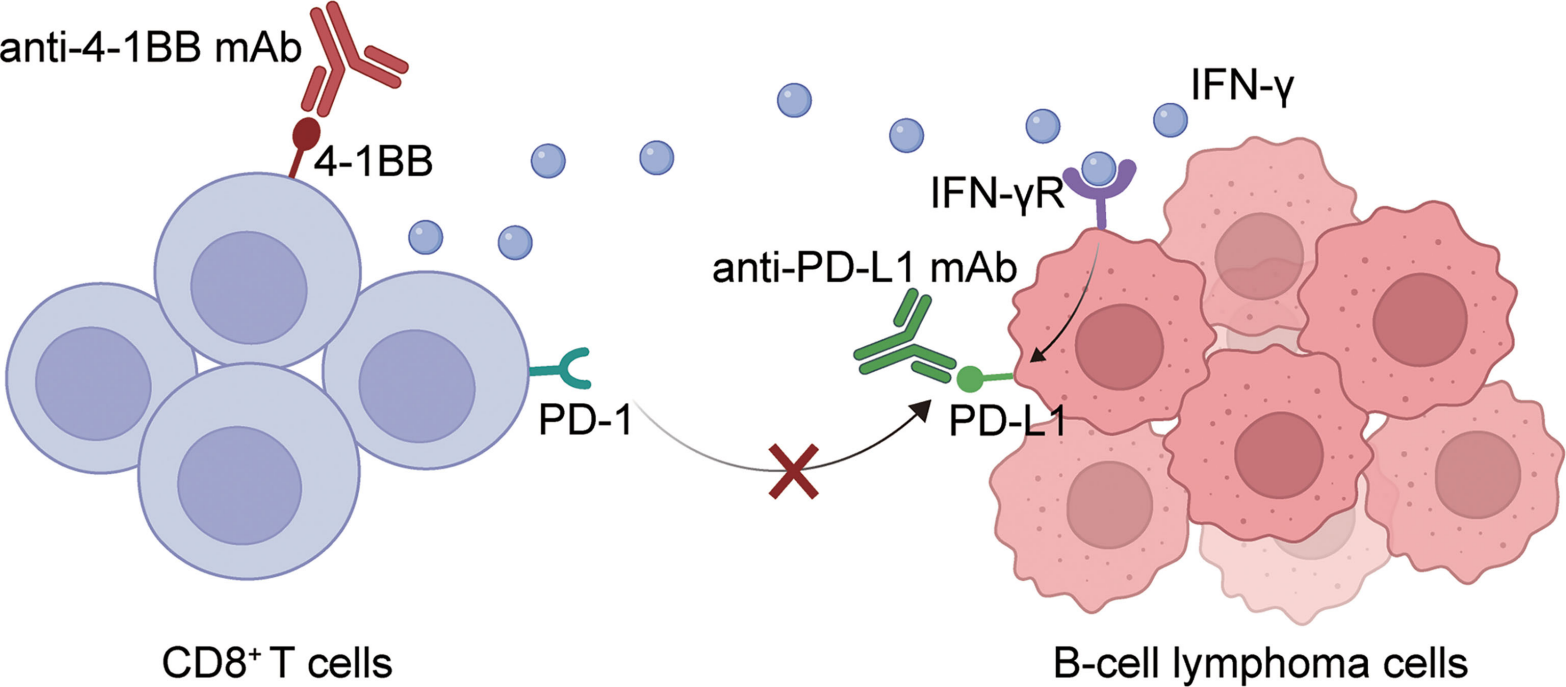
A graphical description of how PD-L1 blockade and 4-1BB agonism elicited enhanced antitumor effect in BCL. The combination of 4-1BB agonist and anti-PD-L1 antibody can activate T cell function and increase the expression of cytokines such as IFN-γ. On the other hand, it can release the inhibition of T cells by tumor cells by blocking the PD-1-PD-L1 signaling axis. Thus, eliciting a more significant anti-tumor effect.

In conclusion, this paper demonstrated that the combination of 4-1BB agonist and anti-PD-L1 antibody can activate T cell function and increase the expression of cytokines such as IFN-γ. On the other hand, it can release the inhibition of T cells by tumor cells by blocking the PD-1-PD-L1 signaling axis. Thus, eliciting a more significant anti-tumor effect (**Figure 6**). Our results confirmed the synergetic antitumor effect of anti-PD-L1 mAb and 4-1BB agonist, providing an effective approach to treating BCL.

## Materials and methods

### Antibodies

Anti-PD-L1 mAb was supplied by Shanghai Hankon Biosciences Co. Ltd (Shanghai, China). 4-1BB agonistic antibody was provided by Eutilex Co., Ltd (Korea). APC anti-CD3 antibody, PE anti-CD8a antibody, and PerCP/Cyanine5.5 anti-CD4 antibody were purchased from BioLegend, Inc.

### Cell culture

A20 and WEHI-231 were purchased from Nanjing Cobioer biotechnology company and cultured in the indicated medium with 10% FBS, and 50 μM B-mercaptoethanol. The cells were incubated at 37 °C and 5% CO_2_.

### *In vivo* therapeutic effect

A20 and WEHI-231 subcutaneous graft tumor models were constructed. The cells were collected by low-speed centrifugation (1200 rpm, 5 min), resuspended in sterile phosphate buffer, and the cell concentration was adjusted to a final concentration of 2.5×10^7^ cells/mL. BALB/c mice were ready for tumor inoculation at six weeks of age, weighing about 20 g. Each mouse was injected with 5×10^6^ cells, as soon as the tumor volume reached 100 mm^3^, the mice were randomly divided into groups and treated. Body weight and tumor diameter (a=long diameter, b=short diameter) were measured and recorded twice a week.


$$\text{Tumor volume}=\frac{a\times b^{2}}{2}.$$

$$\;\text{Relative tumor weight}=100\%\times \frac{\text{control group}-\text{treatment group}}{\text{control group}}.$$

To construct the A20 metastatic tumor model, the cell concentration was adjusted to 1×10^7^ cells/mL using sterile PBS. 100 μL of the cell suspension was injected into the mice through the tail vein, i.e., 1×10^6^ cells/mouse. Treatment was started the day after injection, Mice were treated for one week and administered twice a week (Anti-PD-L1 10mg/kg, 4-1BB agonist 1mg/kg). The survival of mice was monitored.

### RNA-seq

#### RNA extraction

TRIzol® Reagent (Invitrogen) and DNAase I (TaKara) were utilized for total RNA extraction and genomic DNA removal respectively. The quantity and quality of RNA were detected by the ND-2000 (NanoDrop Technologies) and 2100 Bioanalyse (Agilent). The next experiment can only be performed if the RNA meets the following requirements. OD260/280 = 1.8~ 2.2, OD260/230≥2.0, RIN≥6.5, 28S:18S≥1.0, >1μg

#### Library preparation, and Illumina Hiseq xten/Nova seq 6000 sequencing

Firstly, mRNA was isolated from 1 µg of total RNA and fragmented. Then double-stranded cDNA was synthesized and modified with end-repair, ‘A’ base addition and phosphorylation. The 300bp size cDNA was isolated and amplified using PCR. After quantification, sequencing was performed on Illumina Hiseq xten/Nova seq 6000 with read length = 2 × 150bp.

#### Read mapping

SeqPrep and Sickle for quality control. HISAT2 for clean reads aligning to reference genome. StringTie for the assembly of mapped reads.

#### Differential expression analysis and functional enrichment

The expression level was determined by the transcripts per million reads. Q value ≤ 0.05 (DESeq2) was considered to be significantly different. KEGG pathway analysis was carried out by KOBAS.

#### GSEA analysis

GSEA was performed with GSEA v3.0 (http://www.broadinstitute.org/gsea/). Gen sets were obtained from MSigDB (http://www.broadinstitute.org/gsea/msigdb).

### Flow cytometry

#### 4.5.1 Preparation of single cells suspensions of tumor tissues

Mice were executed to obtain tumor tissue. The tumors were cut into small pieces and placed on a cell sieve with PBS for grinding. The collected grinds were centrifuged to remove the supernatant. After 5 min, the supernatant was discarded by centrifugation, washed once with PBS, and PBS was added again to obtain a single-cell suspension.

#### Antibody staining

The supernatant was removed by centrifugation according to the instructions, and the blocking antibody was added and blocked for 30 min at 4°C. The blocking antibody was then removed by centrifugation and the flow-labeled antibody (Anti-mouse CD3 (APC), anti-mouse CD4 (PerCP/Cy5.5), anti-mouse CD8a (PE) (Biolegend)) was added and incubated for a half-hour at 4°C in dark. Centrifuge to remove supernatant, wash twice with PBS, and resuspend in 300μl PBS.

### Immunohistochemistry

Mouse tumors were fixed in 4% paraformaldehyde. After the fixed tissues were embedded in wax blocks and sectioned, the tissue sections were stained using CD 8 antibody. The infiltration of CD8-positive cells in the tissues was examined and analyzed by light microscopy.

### T cells depletion

All antibodies used for T-cell depletion were purchased from bioxcell. Mice were injected intraperitoneally with an anti-CD4 antibody (200μg, colone GK1.5), anti-CD8 antibody (200μg, colone 2.43), and Isotype control (IgG2b, 200μg, colone LTF-2) on days 1, 3, 5, and 7, respectively, and then the antibodies were injected once a week until the end of the experiment.

### Statistical analysis

Data in this study were analyzed by GraphPad Prism 9. Comparison was determined by Student’s *t*-test and One-Way ANOVA analysis, and *P* value< 0.05 was considered as statistical significance.

## Data availability statement

The original contributions presented in the study are publicly available. The data is deposited in the SRA database, accession number: PRJNA904505.

## Ethics statement

The animal study was reviewed and approved by Animal Ethical Committee of School of Pharmacy at Fudan University.

## Author contributions

DJ and XZh designed the study. YW, XZh, CX and YN performed the experiments and wrote the paper. YW, XZh, JF, XZe, and BK analyzed the data. All authors contributed to the article and approved the submitted version.

## Funding

This study was supported by National Natural Science Foundation of China (82073752, 81773620, 81803529, and 32200745), Scientific and Innovative Action Plan of Shanghai (20S11904700 and 20JC1411000) and Shanghai Sailing Program (21YF1401900).

## Conflict of interest

BK is the founder and Chief Executive Officer of Eutilex Co., Ltd.

The remaining authors declare that the research was conducted in the absence of any commercial or financial relationships that could be construed as a potential conflict of interest.

## Publisher’s note

All claims expressed in this article are solely those of the authors and do not necessarily represent those of their affiliated organizations, or those of the publisher, the editors and the reviewers. Any product that may be evaluated in this article, or claim that may be made by its manufacturer, is not guaranteed or endorsed by the publisher.

## References

[1] Chen PL Roh W Reuben A Cooper ZA Spencer CN Prieto PA. Analysis of immune signatures in longitudinal tumor samples yields insight into biomarkers of response and mechanisms of resistance to immune checkpoint blockade. Cancer Discov (2016) 6:827–37. doi: 10.1158/2159-8290.CD-15-1545 PMC5082984 27301722

[2] Neelapu SS Adkins S Ansell SM Brody J Cairo MS Friedberg JW. Society for immunotherapy of cancer (SITC) clinical practice guideline on immunotherapy for the treatment of lymphoma. J Immunother Cancer (2020) 8:e001235. doi: 10.1136/jitc-2020-001235 33361336 PMC7768967

[3] Nishino M Ramaiya NH Hatabu H Hodi FS. Monitoring immune-checkpoint blockade: response evaluation and biomarker development. Nat Rev Clin Oncol (2017) 14:655–68. doi: 10.1038/nrclinonc.2017.88 PMC5650537 28653677

[4] Ayers M Lunceford J Nebozhyn M Murphy E Loboda A Kaufman DR. IFN-gamma-related mRNA profile predicts clinical response to PD-1 blockade. J Clin Invest (2017) 127:2930–40. doi: 10.1172/JCI91190 PMC5531419 28650338

[5] Halstead ES Mueller YM Altman JD Katsikis PD. *In vivo* stimulation of CD137 broadens primary antiviral CD8+ T cell responses. Nat Immunol (2002) 3:536–41. doi: 10.1038/ni798 12021777

[6] Melero I Hirschhorn-Cymerman D Morales-Kastresana A Sanmamed MF Wolchok JD. Agonist antibodies to TNFR molecules that costimulate T and NK cells. Clin Cancer Res (2013) 19:1044–53. doi: 10.1158/1078-0432.CCR-12-2065 PMC4397897 23460535

[7] Choi Y Shi Y Haymaker CL Naing A Ciliberto G Hajjar J. T-Cell agonists in cancer immunotherapy. J Immunother Cancer (2020) 8:e000966. doi: 10.1136/jitc-2020-000966 33020242 PMC7537335

[8] Cohen EEW Pishvaian MJ Shepard DR Wang D Weiss J Johnson ML. A phase ib study of utomilumab (PF-05082566) in combination with mogamulizumab in patients with advanced solid tumors. J Immunother Cancer (2019) 7:342. doi: 10.1186/s40425-019-0815-6 31801624 PMC6894203

[9] Alizadeh AA Gentles AJ Alencar AJ Liu CL Kohrt HE Houot R. Prediction of survival in diffuse large b-cell lymphoma based on the expression of 2 genes reflecting tumor and microenvironment. Blood (2011) 118:1350–8. doi: 10.1182/blood-2011-03-345272 PMC3152499 21670469

[10] Melero I Shuford WW Newby SA Aruffo A Ledbetter JA Hellstrom KE. Monoclonal antibodies against the 4-1BB T-cell activation molecule eradicate established tumors. Nat Med (1997) 3:682–5. doi: 10.1038/nm0697-682 9176498

[11] Demaria O Cornen S Daeron M Morel Y Medzhitov R Vivier E. Harnessing innate immunity in cancer therapy. Nature (2019) 574:45–56. doi: 10.1038/s41586-019-1593-5 31578484

[12] Weiner LM Murray JC Shuptrine CW. Antibody-based immunotherapy of cancer. Cell (2012) 148:1081–4. doi: 10.1016/j.cell.2012.02.034 PMC3310896 22424219

[13] Eskiocak U Guzman W Wolf B Cummings C Milling L Wu HJ. Differentiated agonistic antibody targeting CD137 eradicates large tumors without hepatotoxicity. JCI Insight (2020) 5:e133647. doi: 10.1172/jci.insight.133647 32161196 PMC7141404

[14] Ascierto PA Simeone E Sznol M Fu YX Melero I. Clinical experiences with anti-CD137 and anti-PD1 therapeutic antibodies. Semin Oncol (2010) 37:508–16. doi: 10.1053/j.seminoncol.2010.09.008 21074066

[15] Chen S Fan J Zhang M Qin L Dominguez D Long A. CD73 expression on effector T cells sustained by TGF-beta facilitates tumor resistance to anti-4-1BB/CD137 therapy. Nat Commun (2019) 10:150. doi: 10.1038/s41467-018-08123-8 30635578 PMC6329764

[16] Chester C Ambulkar S Kohrt HE. 4-1BB agonism: adding the accelerator to cancer immunotherapy. Cancer Immunol Immunother (2016) 65:1243–8. doi: 10.1007/s00262-016-1829-2 PMC5035667 27034234

[17] Vinay DS Kwon BS. Immunotherapy of cancer with 4-1BB. Mol Cancer Ther (2012) 11:1062–70. doi: 10.1158/1535-7163.MCT-11-0677 22532596

[18] Hinner MJ Aiba R-SB Wiedenmann A Schlosser C Allersdorfer A Matschiner G. Costimulatory T cell engagement via a novel bispecific anti-CD137 /anti-HER2 protein. J Immunother Cancer (2015) 3:P187. doi: 10.1186/2051-1426-3-S2-P187

[19] Kim YJ Han MK Broxmeyer HE. 4-1BB regulates NKG2D costimulation in human cord blood CD8+ T cells. Blood (2008) 111:1378–86. doi: 10.1182/blood-2007-01-069450 PMC2214739 18024793

[20] Wang Q Zhang J Tu H Liang D Chang DW Ye Y. Soluble immune checkpoint-related proteins as predictors of tumor recurrence, survival, and T cell phenotypes in clear cell renal cell carcinoma patients. J Immunother Cancer (2019) 7:334. doi: 10.1186/s40425-019-0810-y 31783776 PMC6884764

[21] Jeong S Park E Kim HD Sung E Kim H Jeon J. Novel anti-4-1BBxPD-L1 bispecific antibody augments anti-tumor immunity through tumor-directed T-cell activation and checkpoint blockade. J Immunother Cancer (2021) 9:e002428. doi: 10.1136/jitc-2021-002428 34230109 PMC8261887

[22] Tewalt EF Cohen JN Rouhani SJ Guidi CJ Qiao H Fahl SP. Lymphatic endothelial cells induce tolerance *via* PD-L1 and lack of costimulation leading to high-level PD-1 expression on CD8 T cells. Blood (2012) 120:4772–82. doi: 10.1182/blood-2012-04-427013 PMC3520619 22993390

[23] Aznar MA Planelles L Perez-Olivares M Molina C Garasa S Etxeberria I. Immunotherapeutic effects of intratumoral nanoplexed poly I:C. J Immunother Cancer (2019) 7:116. doi: 10.1186/s40425-019-0568-2 31046839 PMC6498680

[24] Liang Y Tang H Guo J Qiu X Yang Z Ren Z. Targeting IFNalpha to tumor by anti-PD-L1 creates feedforward antitumor responses to overcome checkpoint blockade resistance. Nat Commun (2018) 9:4586. doi: 10.1038/s41467-018-06890-y 30389912 PMC6214895

[25] Li X Liu Z Zhang A Han C Shen A Jiang L. NQO1 targeting prodrug triggers innate sensing to overcome checkpoint blockade resistance. Nat Commun (2019) 10:3251. doi: 10.1038/s41467-019-11238-1 31324798 PMC6642086

[26] Parise IZS Parise GA Noronha L Surakhy M Woiski TD Silva DB. The prognostic role of CD8(+) T lymphocytes in childhood adrenocortical carcinomas compared to ki-67, PD-1, PD-L1, and the Weiss score. Cancers (2019) 11:1730. doi: 10.3390/cancers11111730 31694270 PMC6896110

[27] Kim J Choi WS La S Suh JH Kim BS Cho HR. Stimulation with 4-1BB (CD137) inhibits chronic graft-versus-host disease by inducing activation-induced cell death of donor CD4+ T cells. Blood (2005) 105:2206–13. doi: 10.1182/blood-2004-06-2080 15522958

[28] Gomes-Silva D Mukherjee M Srinivasan M Krenciute G Dakhova O Zheng Y. Tonic 4-1BB costimulation in chimeric antigen receptors impedes T cell survival and is vector-dependent. Cell Rep (2017) 21:17–26. doi: 10.1016/j.celrep.2017.09.015 28978471 PMC5645034

[29] Beavis PA Henderson MA Giuffrida L Mills JK Sek K Cross RS. Targeting the adenosine 2A receptor enhances chimeric antigen receptor T cell efficacy. J Clin Invest (2017) 127:929–41. doi: 10.1172/JCI89455 PMC5330718 28165340

[30] He X Feng Z Ma J Zhang X Ling S Cao Y. CAR T cells targeting CD13 controllably induce eradication of acute myeloid leukemia with a single domain antibody switch. Leukemia (2021) 35:3309–13. doi: 10.1038/s41375-021-01208-2 33712688

[31] Peng Q Qiu X Zhang Z Zhang S Zhang Y Liang Y. PD-L1 on dendritic cells attenuates T cell activation and regulates response to immune checkpoint blockade. Nat Commun (2020) 11:4835. doi: 10.1038/s41467-020-18570-x 32973173 PMC7518441

[32] Yang Z Xu J Li L Li R Wang Y Tian Y. Integrated molecular characterization reveals potential therapeutic strategies for pulmonary sarcomatoid carcinoma. Nat Commun (2020) 11:4878. doi: 10.1038/s41467-020-18702-3 32985499 PMC7522294

[33] Williford JM Ishihara J Ishihara A Mansurov A Hosseinchi P Marchell TM. Recruitment of CD103(+) dendritic cells via tumor-targeted chemokine delivery enhances efficacy of checkpoint inhibitor immunotherapy. Sci Adv (2019) 5:eaay1357. doi: 10.1126/sciadv.aay1357 31844672 PMC6905870

[34] Peng DH Rodriguez BL Diao L Chen L Wang J Byers LA. Collagen promotes anti-PD-1/PD-L1 resistance in cancer through LAIR1-dependent CD8(+) T cell exhaustion. Nat Commun (2020) 11:4520. doi: 10.1038/s41467-020-18298-8 32908154 PMC7481212

[35] Liu X Zheng J Sun W Zhao X Li Y Gong N. Ferrimagnetic vortex nanoring-mediated mild magnetic hyperthermia imparts potent immunological effect for treating cancer metastasis. ACS Nano (2019) 13:8811–25. doi: 10.1021/acsnano.9b01979 31328922

[36] Qu QX Zhu XY Du WW Wang HB Shen Y Zhu YB. 4-1BB agonism combined with PD-L1 blockade increases the number of tissue-resident CD8+ T cells and facilitates tumor abrogation. Front Immunol (2020) 11:577. doi: 10.3389/fimmu.2020.00577 32391001 PMC7193033

[37] Kim HD Park S Jeong S Lee YJ Lee H Kim CG. 4-1BB delineates distinct activation status of exhausted tumor-infiltrating CD8(+) T cells in hepatocellular carcinoma. Hepatology (2020) 71:955–71. doi: 10.1002/hep.30881 PMC7154753 31353502

[38] Jeong S Park E Kim HD Sung E Kim H Jeon J. Novel anti-4-1BB×PD-L1 bispecific antibody augments anti-tumor immunity through tumor-directed T-cell activation and checkpoint blockade. J Immunother Cancer (2021) 9:e002428. 34230109 10.1136/jitc-2021-002428 PMC8261887

[39] Emens LA Adams S Cimino-Mathews A Disis ML Gatti-Mays ME Ho AY. Society for immunotherapy of cancer (SITC) clinical practice guideline on immunotherapy for the treatment of breast cancer. J Immunother Cancer (2021) 9:e002597. doi: 10.1136/jitc-2021-002597 34389617 PMC8365813

[40] Galsky MD Balar AV Black PC Campbell MT Dykstra GS Grivas P. Society for immunotherapy of cancer (SITC) clinical practice guideline on immunotherapy for the treatment of urothelial cancer. J Immunother Cancer (2021) 9:e002552. doi: 10.1136/jitc-2021-002552 34266883 PMC8286774

[41] Boyiadzis MM Aksentijevich I Arber DA Barrett J Brentjens RJ Brufsky J. The society for immunotherapy of cancer (SITC) clinical practice guideline on immunotherapy for the treatment of acute leukemia. J Immunother Cancer (2020) 8:e000810. doi: 10.1136/jitc-2020-000810 33077513 PMC7574947

[42] Cheng AN Cheng LC Kuo CL Lo YK Chou HY Chen CH. Mitochondrial lon-induced mtDNA leakage contributes to PD-L1-mediated immunoescape *via* STING-IFN signaling and extracellular vesicles. J Immunother Cancer (2020) 8:e001372. doi: 10.1136/jitc-2020-001372 33268351 PMC7713199

[43] Ansell SM Lesokhin AM Borrello I Halwani A Scott EC Gutierrez M. PD-1 blockade with nivolumab in relapsed or refractory hodgkin's lymphoma. N Engl J Med (2015) 372(4):311–9. doi: 10.1056/NEJMoa1411087 PMC4348009 25482239

[44] Chen R Zinzani PL Fanale MA Armand P Johnson NA Brice P. Phase II study of the efficacy and safety of pembrolizumab for relapsed/refractory classic hodgkin lymphoma. J Clin Oncol (2017) 35(19):2125–32. doi: 10.1200/JCO.2016.72.1316 PMC5791843 28441111

[45] Xu-Monette ZY Zhou J Young. KH. PD-1 expression and clinical PD-1 blockade in b-cell lymphomas. Blood (2018) 131(1):68–83. doi: 10.1182/blood-2017-07-740993 29118007 PMC5755041

[46] Lesokhin AM Ansell SM Armand P Scott EC Halwani A Gutierrez M. Nivolumab in patients with relapsed or refractory hematologic malignancy: Preliminary results of a phase ib study. J Clin Oncol (2016) 34(23):2698–704. doi: 10.1200/JCO.2015.65.9789 PMC5019749 27269947

[47] Green MR Monti S Rodig SJ Juszczynski P Currie T O'Donnell E. Integrative analysis reveals selective 9p24.1 amplification, increased PD-1 ligand expression, and further induction *via* JAK2 in nodular sclerosing hodgkin lymphoma and primary mediastinal large b-cell lymphoma. Blood (2010) 116(17):3268–77. doi: 10.1182/blood-2010-05-282780 PMC2995356 20628145

[48] Armand P Engert A Younes A Fanale M Santoro A Zinzani P. Nivolumab for relapsed/refractory classic hodgkin lymphoma after failure of autologous hematopoietic cell transplantation: extended follow-up of the multicohort single-arm phase II checkmate 205 trial. J Clin Oncol (2018) 36(14):1428–39. doi: 10.1200/JCO.2017.76.0793 PMC6075855 29584546

[49] Herrera AF Moskowitz AJ Bartlett NL Vose JM Ramchandren R Feldman TA. Interim results of brentuximab vedotin in combination with nivolumab in patients with relapsed or refractory Hodgkin lymphoma. Blood (2018) 131(11):1183–94. doi: 10.1182/blood-2017-10-811224 PMC5855021 29229594

