# Supplementary figures and images for: Targeting 4-1BB and PD-L1 induces potent and durable antitumor immunity in B-cell lymphoma
Source: Front Immunol. 2022 Dec 5;13:1004475. doi: 10.3389/fimmu.2022.1004475 (PMC9762552; doi:10.3389/fimmu.2022.1004475)

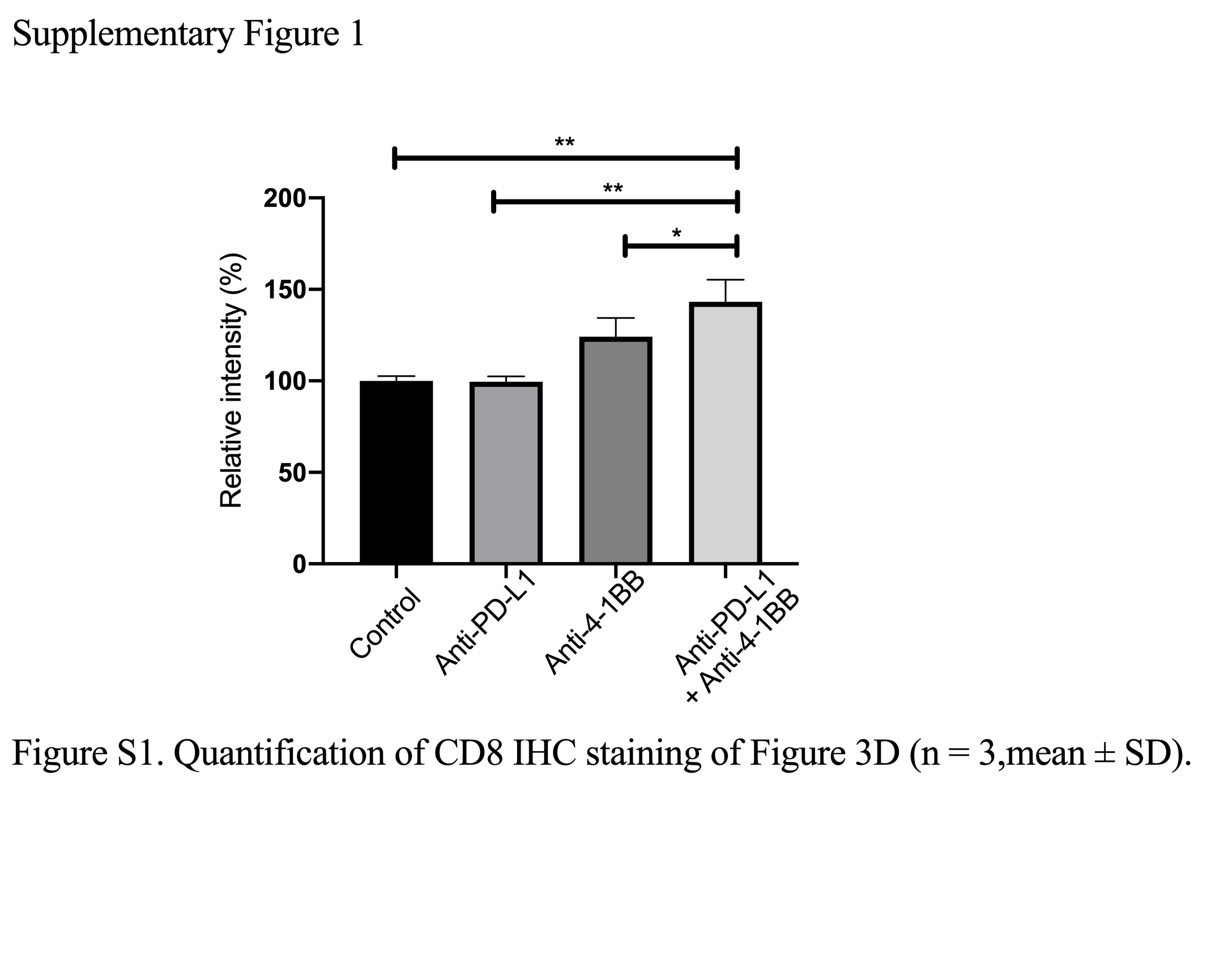

Supplement: Supplementary file 1 [file Image_1.tiff]

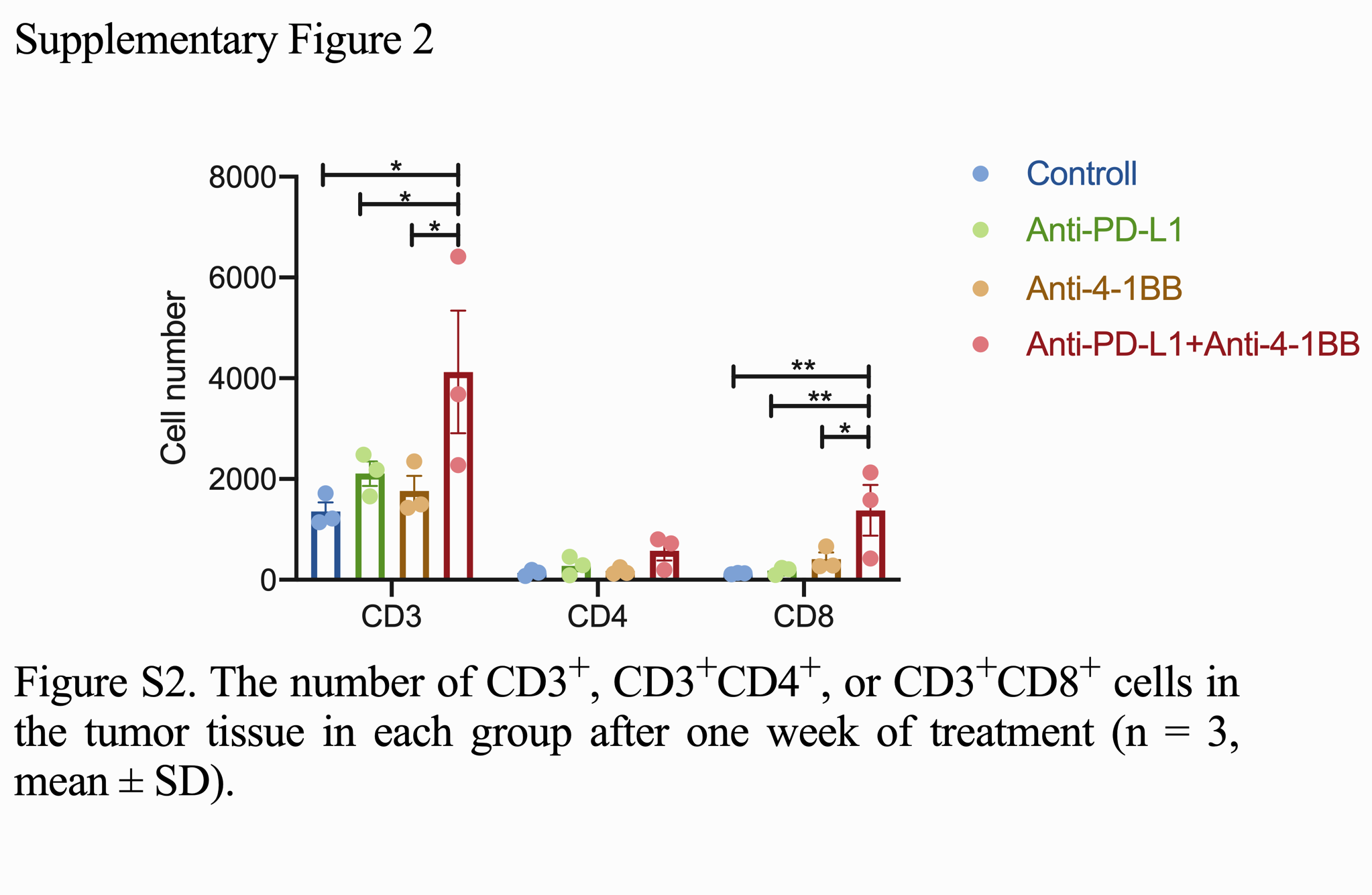

Supplement: Supplementary file 2 [file Image_2.tiff]
